# Spatial landmark detection and tissue registration with deep learning

**DOI:** 10.1038/s41592-024-02199-5

**Published:** 2024-03-04

**Authors:** Markus Ekvall, Ludvig Bergenstråhle, Alma Andersson, Paulo Czarnewski, Johannes Olegård, Lukas Käll, Joakim Lundeberg

**Affiliations:** 1 Science for Life Laboratory, School of Engineering Sciences in Chemistry, Biotechnology and Health, Royal Institute of Technology – KTH, Solna, Sweden; 2 Department of Computer and Systems Sciences, Stockholm University, Kista, Sweden

**Keywords:** Machine learning, Data integration, Transcriptomics

## Abstract

Spatial landmarks are crucial in describing histological features between samples or sites, tracking regions of interest in microscopy, and registering tissue samples within a common coordinate framework. Although other studies have explored unsupervised landmark detection, existing methods are not well-suited for histological image data as they often require a large number of images to converge, are unable to handle nonlinear deformations between tissue sections and are ineffective for *z*-stack alignment, other modalities beyond image data or multimodal data. We address these challenges by introducing effortless landmark detection, a new unsupervised landmark detection and registration method using neural-network-guided thin-plate splines. Our proposed method is evaluated on a diverse range of datasets including histology and spatially resolved transcriptomics, demonstrating superior performance in both accuracy and stability compared to existing approaches.

## Abstract (web summary)

Effortless landmark detection is an unsupervised deep learning-based approach that addresses key challenges in landmark detection and image registration for accurate performance across diverse tissue imaging datasets.

## Main

Spatial landmarks are helpful in various areas of biotechnology. For instance, they are valuable when comparing histological heterogeneity between sites or samples^1^, keeping track of regions of interest in microscopy^2^ or registering tissue samples and transferring them to a common coordinate framework (CCF)^3^. One can obtain spatial landmarks with, for example, experimental labeling^4^, microscopy^2^ techniques and software-based manual or semimanual^5^ annotation. However, the labor-intensive nature of locating spatial landmarks presents a bottleneck in spatial omics data analysis. Automating this process could boost the scalability of sizable spatial omics experiments while obviating the reliance on manually curated annotations.

Researchers have explored automating spatial landmark detection using deep learning techniques in computer vision, with successful results in both supervised^6,7^ and unsupervised settings^8,9^. Unsupervised methods hold greater promise as they can address the general shortage of labeled landmark datasets, particularly in the diverse field of spatial omics. These unsupervised algorithms typically consist of a landmark detector network that identifies landmarks in images and a generative model that uses landmarks to guide image registration.

While these models have shown promise in specific tasks and biological applications^10^, their broad adaptation for tissue-related datasets necessitates overcoming three main challenges: (1) limited datasets: deep learning techniques often necessitate vast datasets, sometimes in the order of 100,000 training examples, to discern general patterns and avoid overfitting. However, multi-omics studies often include fewer than ten training samples. (2) Nonlinear transformations: current methods predominantly focus on datasets involving more straightforward affine transformations, such as rotation, scaling and translation. However, researchers often encounter images that require a combination of elastic and rigid transformations to integrate multiple images in biological contexts^11^. (3) Multimodal data handling: the methods must handle data from different modalities, such as histology stains, spatially resolved transcriptomics and mass spectrometry imaging (MSI), and process these modalities concurrently.

Building on the work of Sanchez et al.^8^ we introduce ELD (effortless landmark detection) to address these challenges, using a landmark detector network for identification and leveraging thin-plate splines (TPSs) for precise image registration without the need for generative modeling. In this study, we highlight the performance of ELD across a range of applications, including single-modality data registration, three-dimensional (3D) modeling and multimodal data alignment. For single-modality data registration, we demonstrate ELD’s enhanced stability and efficiency across modalities such as Visium, hematoxylin and eosin stain (H&E) images and in situ sequencing (ISS). We also show that it outperforms other landmark detection models in numerous tests. Regarding 3D modeling, ELD’s proficiency was underscored by its notable improvement in registration metrics compared to eight other registration models on a mouse prostate dataset. Finally, we show that ELD can successfully model Visium and H&E or Visium and MSI data simultaneously, demonstrating its ability to learn modality-agnostic landmarks for integrating multimodal datasets. Moreover, ELD accomplishes this in an unsupervised manner, eliminating the need for manual annotation and requiring minimal to no parameter tuning, thereby streamlining the process significantly.

## Results

### Benchmarking ELD against existing methods

One can design a deep neural network to better generalize for small training datasets by adding constraints to the model, such as reducing the neuron count per layer, decreasing the number of layers, implementing drop-out or adding a regularizer to the loss function^10^. In ELD, we constrain the solution space by removing the generative network while retaining the landmark-detecting network, as suggested by Sanchez et al.^8^. With the image landmarks identified, registration can be easily performed using landmark-based methods, such as homography^11^ or TPSs^12^. However, given the elastic nature of the transformations in our data, we here use TPS for registration purposes. These methods offer several advantages, including having fewer unlearnable parameters (hard constraints) and being more computationally efficient than large deep neural networks.

The fundamental distinction between ELD and previous methodologies lies in their approach to alignment. Traditional methods use a generative deep neural network for alignment, which, in the context of small datasets, leads to the generation of seemingly random and inconsistent landmarks. These landmarks, essentially serving as identifiers, can be memorized by the generative network, enabling it to align images, but with landmarks that lack meaningful correspondence or consistency. ELD, on the other hand, employs the analytically solvable TPS. This approach prevents the landmark detector from creating image-specific identifications as TPS lacks memorization capabilities. Consequently, this compels the landmark detector to identify consistent landmarks across tissue sections. Additionally, while previous methods are typically limited to pairwise image alignment with linear noise, ELD expands these capabilities. It can handle more complex scenarios such as high-dimensional data (such as Visium), 3D alignment and multimodal data, all of which necessitate new and more sophisticated optimization heuristics that are thoroughly explained in the Methods section.

**Fig. 1:**
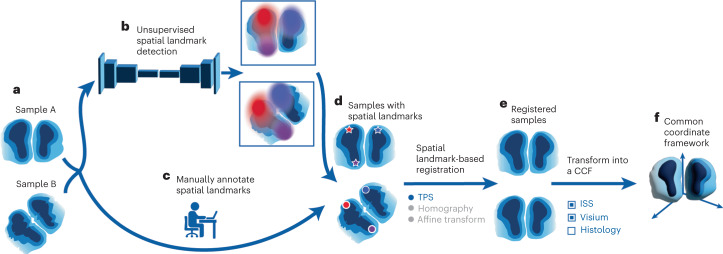
Overview of ELD framework. Proposed workflow of acquiring spatial landmarks and aligning sections within a CCF. The process can be divided into steps as follows. **a**, Obtain two different sections, A and B, for which we want to identify landmarks. **b**,**c**, Use a trained unsupervised spatial landmark detection network (**b**) or manually annotate the sections to obtain landmarks for both sections (**c**). **d**, After acquiring landmarks for both samples, register Section B to Section A using various landmark-based alignment methods, such as TPS or homography. **e**, Obtain the registered sample, which aligns Section B with Section A. **f**, Map the samples to a CCF, allowing cross-section comparisons and analysis.

The process of aligning tissue slices to a CCF can be outlined as follows (Fig. 1): to begin, the ELD system uses an unsupervised trained spatial landmark detection network to pinpoint landmarks on the desired tissue slices or manual annotations can be used. Once these critical points have been established across all slices, ELD uses landmark-centric alignment techniques, such as TPS or homography, to align the regions. As a final step, ELD projects all the aligned tissue regions onto a CCF, facilitating comparative studies across various slices.

In this study, we use standard error metrics to assess the performance of ELD and two other state-of-the-art landmark detection methods. These metrics include forward error, backward error and consistency error^8^. Performance benchmarks are conducted using the CelebA dataset for training, and the MAFL and AFLW datasets for evaluation, which are frequently used for tasks of this nature.

**Fig. 2:**
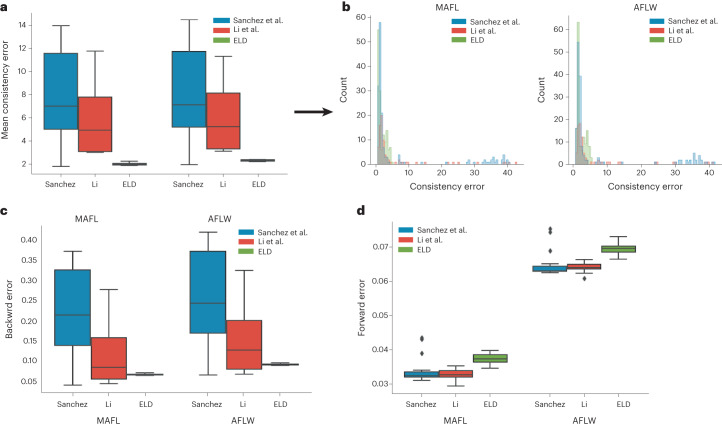
Performance benchmarks for ELD. **a**–**d**, The performance benchmarks for ELD and two other state-of-the-art models for landmark detection. Benchmarks were conducted on the MAFL and AFLW datasets with 1,000 and 2,991 samples, respectively. The parts show the distribution of the mean consistency error for each image (**a**), consistency error for each landmark (**b**), and backward (**c**) and forward errors (**d**) for each image. The box plots display the median (central line within each box), the interquartile range (boxes representing the 25th to 75th percentiles) and the whiskers extend to 1.5 times the interquartile range above and below the box.

Consistency error evaluates landmark stability through geometric consistency. To calculate the consistency error, one must: (1) detect the landmarks in the image, apply an affine transformation to the landmarks and (2) apply the same affine transformation to the image and then detect the landmarks again but on the transformed image. The error is determined by comparing the point-to-point distances between the two sets of landmarks. ELD exhibits superior consistency compared to other methods (Fig. 2a). A closer examination of the results reveals that the performance difference is largely attributed to the other methods’ tendency to identify landmarks that are sometimes significantly misaligned (Fig. 2b). While most landmarks are consistent, these outliers contribute to a much higher mean error than ELD.

The forward error is calculated by training a linear regression model using a set of manually annotated points and the detected landmarks. The trained regressor predicts the annotated points based on the detected landmarks. Conversely, the backward error is computed using a linear regression model trained in reverse order: that is, using the annotated landmarks to predict the detected landmarks. This error serves as a measure of the stability of the detected landmarks. A model with a low forward error but a high backward error will likely detect a low number of stable landmarks. On the other hand, a model that has a low backward error but high forward error is likely to converge to a fixed set of points independent of the input image.

ELD exhibits significantly better backward error than other methods (Fig. 2c), which can be attributed to the inconsistent landmarks found by the other methods. Although all models show better performance in forward error than backward error, ELD displays marginally worse performance in forward error (Fig. 2d). This indicates that ELD sacrifices some generalization in favor of significantly improved consistency.

**Fig. 3:**
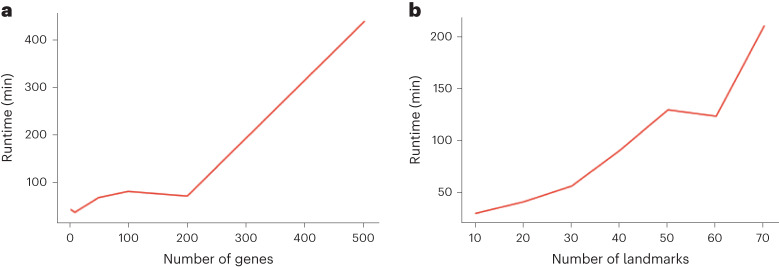
Runtime analysis. **a**,**b**, The time required for convergence in minutes as a function of the number of genes or color channels used when the number of landmarks is set to ten (**a**) and as a function of the number of landmarks used when the number of channels is set to three (**b**).

We conducted two tests to evaluate ELD’s runtime requirements: one with varying numbers of genes or image channels (Fig. 3a) and another with varying numbers of landmarks (Fig. 3b). As detailed in the Methods section, the convergence criterion is stringent; however, convergence is typically achieved more quickly in real-world applications.

### Performance evaluation on single-modality data

**Fig. 4:**
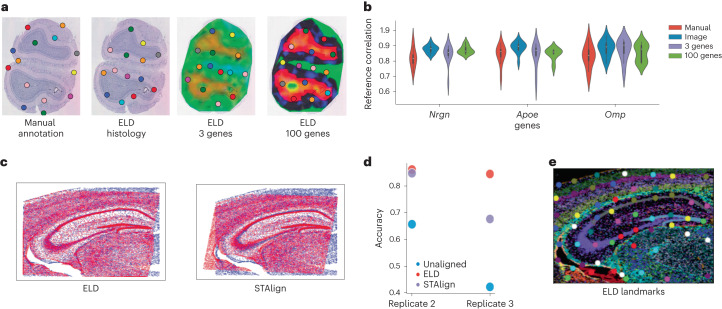
Performance on single-modality data. **a**, Landmarks identified by ELD when trained on various modalities, such as histology and Visium data, alongside an image with manual annotation for comparison. The rightmost image, which includes 100 genes, is visualized using PCA; however, all genes were used during the training process. For all experiments, 14 landmarks were used. **b**, Violin plots show the correlation of three target genes (*Nrgn*, *Apoe* and *Omp*) between the ten registered samples and the reference. The violin plots display the median (white dot within each box inside the violin), the interquartile range (boxes representing the 25th to 75th percentiles) and the whiskers extend to 1.5 times the interquartile range above and below the box. **c**, Visual comparison of registration quality between ELD and STAlign for ISS data. **d**, Evaluation of how accurately a *k*-nearest neighbor model predicts different anatomical regions with three samples. **e**, ELD-predicted landmarks on an RGB image generated by clustering the ISS data.

An effective registration method for Visium data, Eggplant^13^, is currently openly available. One limitation of Eggplant, however, is its reliance on manual spatial landmark annotation. Therefore, we next sought to test whether the spatial landmarks generated by ELD can supplant manual annotation and improve the performance of Eggplant. Using Eggplant to transfer the gene expression of three target genes with distinct expression patterns (*Nrgn*, *Apoe* and *Omp*) in the mouse olfactory bulb to a reference section using either manually or automatically detected landmarks, we find that the landmarks produced by ELD yield results that are at least as accurate as those obtained using manual annotation (Fig. 4a,b). For this experiment, we used 12 mouse olfactory bulb samples, the same reference as used in Eggplant^13^. Our results are consistent whether landmarks were identified using histology or expression data from three or 100 genes.

We used three mouse brain coronal sections from Salas et al.^14^ to demonstrate ELD’s compatibility with ISS data. In this experiment, we use RGB (red, green, blue) images of the clustering on the ISS data (Fig. 4e) and use TPS for the final registration. To evaluate the effectiveness of the registration, we assess how well a simple *k*-nearest neighbor model trained on the reference could predict the correct anatomical region on the registered samples. Comparing ELD to STAlign^15^, which has shown promising results for aligning data from ISS experiments, we find that ELD attains a higher accuracy in both replicates (Fig. 4d).

### 3D modeling

**Fig. 5:**
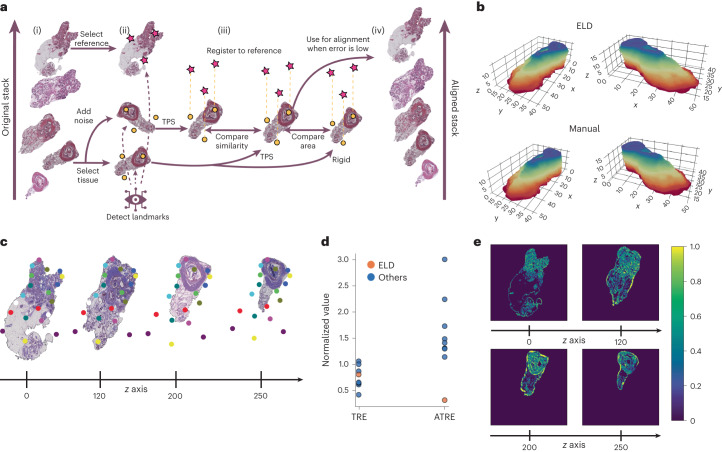
3D tissue reconstruction with ELD. **a**, Illustration of the 3D modeling process. (i) Initially, select a reference image at random from the tissue stack, choose a specific tissue and create a counterpart with introduced deformations to map onto the reference. This step involves generating a deformed version of the source tissue to simulate variations or distortions. (ii) Next, detect landmarks for both the reference image and the source tissue, including its deformed version. Finally, map the deformed source image to the reference using TPS, and then align the original source image with the reference using both TPS and a rigid transformation technique. (iii) Compute the similarity loss between the mapped noisy source and the original source image, and compare the area change between the source image mapped with TPS and rigid transformation. Repeat this process for all tissues in the stack until convergence. (iv) Finally, map all tissues to the reference. **b**, Demonstration of final registration using ELD, and with the manual annotations for 260 prostate samples. **c**, Display of aligned tissues with their anchors from the tissue stack. A total of 20 landmarks were used for the alignment. **d**, Performance comparison of ELD and other models based on the 260 prostate samples. All results are normalized using the value obtained when aligning with manual landmarks (corresponding to a score of 1). **e**, Absolute error between manual alignment and alignment using ELD across four sections in the *z*-stack.

To make it possible to align a stack of multiple tissue sections, whose morphology may change drastically along the stacking axis, we modify ELD to generate anchor points instead of landmarks. The general procedure is illustrated in Fig. 5a. Briefly, the most significant difference for *z*-stack alignment involves controlling how the area changes of the transformed tissue. This forces the landmarks to act more like anchor points with fixed *xy* coordinates instead of identifying common morphology, as demonstrated in Fig. 5c.

To assess ELD’s 3D alignment performance, we use a mouse prostate dataset containing 260 slices from Kartasalo et al.^16^. The dataset contains annotations from two different annotators of four corresponding landmarks in each pair of consecutive sections. Additionally, we use their published code to generate the results. Since most benchmarks were similar across all methods and different processing was done on the images, the root-mean-square error (r.m.s.e.) is difficult to compare fairly. Therefore, we chose to only present the landmark-related benchmarks target to registration error (TRE) and accumulated TRE (ATRE). The TRE is calculated as the Euclidean distance between the actual and predicted locations of points. Specifically, these points are not used in the registration process (also known as target points), and this calculation is performed for each consecutive pair. The ATRE is a cumulative TRE over all the tissue sections. This measurement provides an overall indication of the total error in the registration task across all target points. The mean of TRE and ATRE is used, and it is normalized by the score obtained when registering with manually annotated landmarks, as depicted in Fig. 5d. While the performance of ELD is comparable to the other methods in terms of TRE, ELD significantly outperforms them in terms of ATRE, suggesting that the alignment is more consistent across the entire tissue volume. We compared eight other registration models, seven of which come from Kartasalo et al.^16^ and CODA^17^. The final 3D alignment is illustrated in Fig. 5b.

### Performance evaluation on multimodal data

**Fig. 6:**
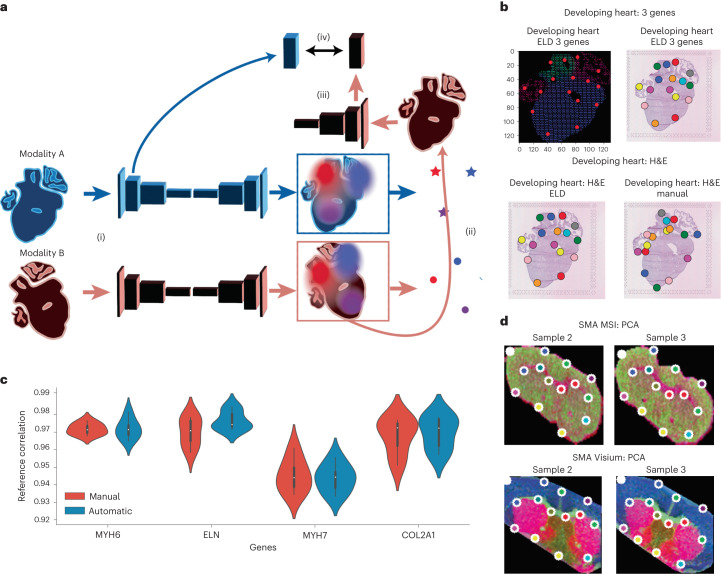
Benchmarking ELD for multimodal alignment. **a**, Registration of multimodal data, overview. (i) Each modality, A and B, is passed through its respective landmark detector, and a latent representation of modality A is saved. (ii) Landmarks are identified within the heatmaps by pinpointing the pixel with the highest intensity for each individual heatmap, a process known as the spatial argmax operation. Following this, the samples from the two modalities are registered. (iii) A latent representation is obtained from modality B. (iv) We now aim to maximize the similarity between the latent representations of modalities A and B. This optimization is a crucial part of the training process, where two key objectives need to succeed: first, ensuring the landmark detector identifies corresponding landmarks across modalities, leading to accurate registration; and second, embedding both modalities into a joint latent space. By minimizing the distance of the modalities’ latent representations into the loss function, we progressively refine our two objectives during the training process. **b**, Detected landmarks for gene expression and histology images. The upper-left image shows *MYH6*, *ELN* and *MYH7* gene expression. In total, 16 landmarks were used for this experiment. **c**, Performance comparison of Eggplant’s alignment using manually annotated landmarks and landmarks detected with ELD with three samples (three registered samples to one target sample). The violin plots display the median (white dot within each box inside the violin), the interquartile range (boxes representing the 25th to 75th percentiles), and the whiskers extend to 1.5 times the interquartile range above and below the box. **d**, PCA visualization of MSI and Visium data with their respective landmarks.

ELD can detect landmarks and align tissue data from different modalities. To optimize the alignment between two distinct modalities, separate landmark detectors are used for each modality. During training, random samples from both modalities are selected, one sample is registered to the other and their alignment is assessed in the latent space obtained from the landmark detector (Fig. 6a).

We used the Human Developing Heart dataset^13^, which consists of four samples, to demonstrate ELD’s ability to align tissues from two different modalities. Histology images were used for the first two samples, while the genes *MYH6*, *ELN* and *MYH7* from Visium expression data were used to construct an image for the other samples. The detected landmarks for the two modalities are displayed (Fig. 6b).

To benchmark ELD’s performance, we randomly selected one of the samples as reference. Then we calculated the correlation of the source sample to the reference with Eggplant, using both ELD’s landmarks and manually annotated landmarks. The programmatically detected landmarks perform comparably to manually annotated landmarks (Fig. 6b,c).

To further demonstrate the flexibility of ELD to model data of diverse modalities, we apply it to principal component analysis (PCA) embeddings of MSI and Visium data. This data was extracted from three mouse striatum samples as per the study conducted by Vicari et al.^18^. For each of these samples, we used a combination of both MSI and Visium methodologies. We find the generated landmarks to be qualitatively consistent across sections and to mark out biologically relevant anatomical features (Fig. 6d).

## Discussion

In this study, we have introduced ELD, a method for unsupervised spatial landmark detection and registration that addresses the challenges of small datasets and nonlinear transformations typically found in spatial omics and histological image data. ELD employs neural-network-guided TPSs and outperforms existing approaches in terms of both accuracy and stability.

By removing the generative network and retaining the landmark-detecting network, ELD effectively addresses the issue of overfitting in small training datasets by removing the generative network and retaining the landmark-detecting network. We have demonstrated that ELD achieves superior consistency and backward error performance compared to competing methods while showing marginally worse performance in forward error. Our runtime tests indicate that ELD is computationally efficient, and we have empirically observed convergence to be even quicker in many real-world applications.

By tweaking the optimization function, we have demonstrated the effectiveness of ELD in a wide range of applications, such as single-modality data registration, 3D modeling and multimodal data alignment. Regarding single-modality data registration, ELD outperformed existing methods such as manual annotation with Eggplant and STAlign. For 3D modeling, ELD was adapted to produce anchor points rather than landmarks, leading to successful *z*-stack alignment. Moreover, ELD significantly improved the ATRE metric for the mouse prostate dataset compared to eight competing models.

Finally, we have shown that ELD can align tissues from diverse modalities by using distinct landmark detectors for each modality and comparing the registration similarity in a latent tissue space.

ELD’s capability to learn landmark detection across different modalities, both in unimodal and multimodal settings, effectively addresses the challenges of homogeneity often encountered in H&E tissues with limited anatomical structure. In such cases, ELD often identifies landmarks predominantly around the tissue borders. While this leads to satisfactory registration results, the identified landmarks might lack significance or interest. To enhance the landmark detection and achieve more meaningful landmarks, introducing additional modalities such as MSI or Visium is beneficial. These modalities usually exhibit more heterogeneous textures, providing a richer context for ELD to detect varied and significant landmarks. In scenarios where only a single modality with very homogeneous structures is accessible, and there is a desire to identify more intriguing landmarks, investigating alternative landmark detection techniques can be advantageous. Approaches such as the oriented fast and rotated BRIEF, developed by Maric et al.^19^, may offer potential improvements. However, it is important to note that in our experiments, oriented fast and rotated BRIEF-based methods did not yield satisfactory results with our datasets, leading us to not include them. This shows the variability in the effectiveness of different techniques depending on the specific characteristics of the analyzed data.

The primary objective of ELD is landmark detection, while registration serves as an added benefit. In this regard, relatively simple registration models, such as TPS, have been used. We believe that ELD has the potential to improve other models with more advanced registration approaches, such as STAlign and CODA, similar to how it enhances Eggplant, by supplying ELD’s landmarks as a ground truth during the training phase.

Overall, ELD demonstrates a notable improvement over existing unsupervised landmark detection and registration methods in spatial omics and histological image data. Its versatility in addressing different data types and modalities makes it a promising tool for researchers in spatial biology.

## Methods

### Hardware

We used an NVIDIA A100-SXM 81GB graphics card and 12 AMD EPYC 7742 64-core processors for all model training.

### Cost function from MS-SSIM

In all the experiments detailed in the subsequent sections, we use a cost function rooted in the multiscale structural similarity (MS-SSIM) method. This approach allows for a comprehensive assessment of image quality by considering image details across a range of resolutions. This method extends the single-scale SSIM index, which compares luminance, contrast and structure of two aligned signals, such as image patches^20^. MS-SSIM has been very useful in our experiments since we have due to the presence of significant batch effects, and MS-SSIM have demonstrated more robustness than, for instance, mean squared error in our experiments.

The MS-SSIM procedure involves an iterative process of applying a low-pass filter to the image and downsampling the filtered image. Each iteration defines a new scale, culminating in the highest scale. Contrast and structure comparisons are computed at every scale, while luminance comparison is reserved for the highest scale^20^.

The overall quality assessment in MS-SSIM combines these measurements from all scales, using adjustable parameters for accounting for the relative importance of each component at every scale. The method yields a detailed image quality map, with the mean MS-SSIM index offering an overall evaluation of image quality. For a comprehensive understanding of MS-SSIM, refer to the work of Wang et al.^20^.

For the calculation of MS-SSIM, we used the PyTorch Image Quality Assessment package, using its default parameters along with a window size of five. This configuration was chosen based on our preliminary trials, which indicated its effectiveness in our context.

### Landmark drop-out

When training ELD landmarks, they can become trapped in a local minimum, which often results in many landmarks occupying similar positions. To counteract this, we use a technique known as landmark drop-out. This process involves the probabilistic removal of detected landmarks, with each landmark having a probability *P* of being dropped out. Empirical observations have demonstrated that this method allows the landmarks to escape local minima more rapidly, leading to a more diverse and satisfactory distribution of landmarks in a shorter time. Throughout all our experiments, we used a drop-out probability of 10%. Empirically, this has proven to work well in practice.

### Cropping

During training, data augmentation can occasionally cause images to be cut off or contain missing regions due to batch effects. This can complicate the process when registering two images, as the missing portions can confuse the model. To mitigate this issue, we perform a cropping procedure on the registered and mapped images based on the black-colored background, which is represented by a zero value across all channels. Specifically, we identify the masks for all black channels in both images and then crop both images according to these masks. To ensure precision, we implement a threshold of 0.1. This means any pixels in which all channels fall below this threshold are considered black.

### Registration using TPSs

In all the experiments outlined in the following sections, we use TPS for image registration. TPS is a widely used method known for its capacity to effectively handle image registration and deformation, primarily by interpolating scattered data points. TPS creates a smooth and flexible mapping between two sets of landmarks while minimizing bending energy. Formally, consider a set of *n* source points ${p}_{i}$ and their corresponding target points ${q}_{i}$ in a two-dimensional space. The objective is to determine the bias parameter ${a}_{1}$, the affine parameters ${a}_{x}$ and ${a}_{y}$, and the nonlinear parameters ${w}_{i}$ for each of the *x* and *y* coordinates. These parameters should be optimized such that the mapping function $f(x,y)$ minimizes the following energy function.

$$U(r)={r}^{2}{\log }_{2}(r)$$

$${f}_{x}(x,y)={a}_{1}+{a}_{x}x+{a}_{y}y+\mathop{\sum }\limits_{i=1}^{N}{w}_{i}U({\rm{||}}({x}_{i},{y}_{i})-(x,y){\rm{||}})$$

$${f}_{y}(x,y)={a}_{1}+{a}_{x}x+{a}_{y}y+\mathop{\sum }\limits_{i=1}^{N}{w}_{i}U({\rm{||}}({x}_{i},{y}_{i})-(x,y){\rm{||}})$$

The TPS function, $f(x,y)$, can be solved analytically to obtain the weights. Once these weights are acquired, the source image can be mapped to the reference. For a more in-depth understanding, we recommend referring to the study by Keller et al.^12^.

TPS is of significant utility in scenarios demanding smooth and continuous transformations, such as shape morphing in computer graphics. Within the context of ELD training, TPS is leveraged to guide the learning of high-quality landmarks.

### Landmark detector

The landmark detector used in this article is identical to the one used by Sanchez et al.^8^, which is an hourglass network consisting of approximately 6 million parameters.

### Neural-network-guided TPSs landmark detection

The ELD framework primarily consists of two components: (1) a deep neural network for landmark detection, and (2) TPS for image registration. The landmark detector processes the source and target images to identify a set of source points ${p}_{i}\in P$ and corresponding target points ${q}_{i}\in Q$. We then fit the parameters of the TPSs by determining a function $f$ such that *f*(*P*) *=* *Q*. This function $f$ is subsequently used to warp the source image to align with the target image.

Initially, however, the landmark detector lacks an inherent understanding of what constitutes landmarks, often resulting in the generation of arbitrary points for *P* and *Q*. Consequently, the parameters for the TPSs, based on these random landmarks, lead to inaccurate registration. To address this, we refine the landmark detector using a training process that minimizes the loss, defined as the dissimilarity between the target image and the warped source image. This training approach encourages the detector to identify corresponding landmarks in both the source and target images, essential for successful registration with the TPSs. As the training progresses, the landmark detector gradually improves, learning to identify more accurate and correspondingly relevant landmarks, thereby enhancing the overall registration accuracy.

### General image preprocessing

Throughout all experiments, we used 128 × 128 images during training, achieved by using cv2.resize and applying INTER_AREA-interpolation (referenced in docs.opencv.org) to transform the original image dimensions to the desired format. Furthermore, ELD cannot process flipped images, so it is crucial to ensure all images are oriented in the same way before training.

### Data augmentation

In every experiment, we used data augmentation strategies, including rotation, scaling and elastic transformation. The rotation was implemented with a random angle selection between −15 and 15°, paired with appropriate scaling to maintain the participant within the image frame. For the introduction of elastic noise, we employed the elasticdeform package. Both the control points for the deformation grid and the sigma for the normal distribution were randomly selected within an experiment-dependent range.

### Visium preprocessing and filtering

For all Visium data, spots with fewer than 200 detected genes were removed, and genes present in fewer than three spots were also removed. The data were then normalized using Scanpy and log_1*p*_-transformed. The same genes as in Eggplant^13^ were chosen when selecting three genes. In the experiments where more genes were used, we performed Leiden clustering on neighborhood graphs derived from PCA. All methods were implemented with the default parameters provided by Scanpy. Subsequently, we used Scanpy’s rank_genes_groups function, using the Leiden clusters as groupings and the *t*-test for ranking. This allowed us to select the top *n*-ranked genes per sample. Finally, the common genes across all samples were chosen for further analysis.

To adapt the Visium data for compatibility with ELD, it was necessary to normalize the gene expression values to a range between 0 and 1. We used Scipy’s interpolate.griddata function with linear interpolation to convert this data into continuous images. This method allows us to predict the values of intermediate pixels between the spots accurately.

Regarding the visualization presented in Fig. 2b, for Visium data comprising three genes, we treated the data as if it were RGB images, using the gene expression data directly for visualization. For the Visium data encompassing 100 genes, a different approach was required. We treated all pixels as individual samples and used Scikit-learn’s PCA from decomposition.PCA. This enabled us to distill the data into three principal components. Consequently, we could transform the expression data of all 100 genes for each pixel into these three principal components, facilitating an effective visualization of the complex gene expression patterns.

### Comparative assessment of landmark quality and runtime requirement: evaluating existing methods

During the training of ELD on the CelebA dataset for the purpose of landmark quality assessment, we create two augmented variants of each image: *X*_target_ and *X*_source_, as elaborated in the preceding section. *X*_source_ is then registered to *X*_target_ using the landmarks detected by the ELD, resulting in *X*_registered_. We then calculate the MS-SSIM loss between *X*_registered_ and *X*_target_, which is referred to as the base loss.

$${L}_{\mathrm{base}}={\mathrm{MS-SSIM}}\left({X}_{\mathrm{registered}},{X}_{\mathrm{target}}\right)$$

To guarantee consistency across images, we randomly select another sample, *X*_target_, and align it to *X*_target_, forming *Y*_registered_. However, we only compute the MS-SSIM loss (with a window size of 3) between small patches surrounding the landmarks, specifically ${P}_{{Y}_{\mathrm{registered}}}$ and ${P}_{{X}_{\mathrm{target}}}$. This is called the consistency loss and ensures that specific landmarks, such as the left eye, consistently target the same feature across different images.

$${L}_{\mathrm{consistency}}={\mathrm{MS-SSIM}}\left({P}_{{Y}_{\mathrm{registered}}},{P}_{{X}_{\mathrm{target}}}\right)$$

The primary loss is computed by combining the base loss and the consistency loss. The consistency loss is scaled by a factor of 0.1, determined through empirical testing, although a scalar in the range of 0.1 to 0.5 has been observed to yield similar performance. The final loss function is, therefore, a composite of these two components.

$${L}_{\mathrm{total}}={L}_{\mathrm{base}}+0.1 \times {L}_{\mathrm{consistency}}$$

In this comparison, we evaluated our method against two other recently published landmark detection methods^8,9^, both of which represent the current state of the art in this field. We trained a total of 15 models for each method. We ran their models using default parameters for 80 epochs, a batch size of 48 and also trained our models with the same parameters, but added elastic noise with a sigma value of 3. The learning rate used was 1 × 10^−4^, using a learning rate scheduler with a step size of ten epochs and a learning rate decay of 0.95. TPS was used to register the samples. All benchmarks were performed with the code from Sanchez et al.^8^.

In the runtime experiment, we used an initial learning rate of 1 × 10^−4^, which was annealed by a learning rate scheduler with step size 3 and a learning rate decay of 0.95. TPS was used for registration purposes. Samples were perturbed by elastic noise with a sigma parameter of 5.5. A batch size of 48 was used, resulting in 300 iterations per epoch. Training was stopped when the loss improved by less than 1 × 10^−4^ over ten consecutive epochs.

### Performance evaluation on single-modality data

Maintaining the same objective outlined in the preceding section, but we modified the calculation of *L*_consistency_. Instead of applying MS-SSIM to patches of the aligned sections, we computed it directly between *X*_target_ and *Y*_registered_.This adjustment is justified given the presence of minor batch effects, which represent technical variations among the samples. Consequently, the consistency loss, denoted as *L*_consistency_, is calculated as the MS-SSIM between *Y*_registered_ and *X*_target_:

$${L}_{\mathrm{consistency}}={\mathrm{MS-SSIM}}\left({Y}_{\mathrm{registered}},{X}_{\mathrm{target}}\right)$$

The final loss, *L*_total_, is then computed by combining the base loss, *L*_base_, with *L*_consistency_, where the latter is scaled by a factor of 0.1:

$${L}_{\mathrm{total}}={L}_{\mathrm{base}}+0.1 \times {L}_{\mathrm{consistency}}$$

We adhered to the same learning rate schedule, perturbation parameters, batch size and stopping criteria as delineated in the section discussing runtime experiments. All Visium data was preprocessed following the methodology outlined in the preceding section.

When comparing with STAlign, we use the same default parameters outlined in their article^15^. The process began with annotating each image with three distinct landmarks, details of which can be found in the Supplementary Fig. 1. Using STAlign’s L_T_from_points function, we calculated the affine transformation between the source and target images.

Subsequently, we applied STAlign’s LDDMM function, configuring it with the following parameters: niter (number of iterations), sigmaM, sigmaA, sigmaB and epV. The values assigned to these parameters were 300, 0.15, 0.1, 0.11 and 10, respectively.

The final step involved using STAlign’s transform_points_target_to_atlas function to execute the transformation.

### 3D modeling

During the training process, for each individual sample *X*_*i*_ drawn from the complete stack ${X}_{1},\ldots ,{X}_{N}$, where *N* is the total number of sections, a random reference point *X*_reference_ is chosen from the z-stack. Furthermore, an additional sample, *X*_*j*_, is selected at random from within the range ${X}_{i-3}$ to ${X}_{i+3}$, with a certain amount of noise introduced. Landmarks are identified for each sample in this triplet: *X*_reference_, *X*_*i*_ and *X*_*j*_.

The nondistorted *X*_*i*_ is registered to *X*_reference_ using both a rigid transformation (using the Kabsch–Umeyama algorithm) and a TPS transformation, resulting in two different versions of the registered image, denoted as ${X}_{i}^{\mathrm{TPS}}$ and ${X}_{i}^{\,\mathrm{rigid}}$. The noisy variant *X*_*j*_ is registered to the reference point using TPS, referred to as ${X}_{j}^{\mathrm{TPS}}$.

Subsequent to registration, we compute the change in area, d*A*, before and after registration with TPS between *X*_*i*_ and ${X}_{i}^{\mathrm{TPS}}$. The loss function is then given as:

$$L=(1-{{\mathrm{d}}A}) \times {\mathrm{MS-SSIM}}\left({X}_{j}^{\mathrm{TPS}},{X}_{i}^{\mathrm{TPS}}\right)+{{\mathrm{d}}A} \times {\mathrm{MS-SSIM}}\left({X}_{j}^{\mathrm{TPS}},{X}_{i}^{\mathrm{rigid}}\right)$$

In this loss function, the first part is the product of MS-SSIM calculated between ${X}_{i}^{\mathrm{TPS}}$ and ${X}_{j}^{\mathrm{TPS}},$ and (1 − d*A*). The second part is the product of MS-SSIM calculated between ${X}_{i}^{\,\mathrm{rigid}}$ and ${X}_{j}^{\mathrm{TPS}}$, multiplied by d*A*.

This means that if the area changes significantly after registration, ${X}_{i}^{\mathrm{rigid}}$ contributes more to the loss function, which could lead to a less optimal fit. This strategy compels the landmarks to function more as anchor points, ensuring increased stability throughout the *z*-stack.

We trained the model for 80 epochs, a batch size of 258 (the whole *z*-stack), resulting in 300 iterations per epoch, and with elastic noise with a sigma value of 5.5. The learning rate used was 1 × 10^−4^, using a learning rate scheduler with a step size of ten epochs and a learning rate decay of 0.95.

All benchmark metrics were performed with the code from Kartasalo et al.^16^.

### Performance evaluation on multimodal data

In the same way as in previous sections, we calculate a base loss by identifying landmarks and registering a sample with a noisy variant of itself. However, when we deal with multimodal data alignment, each data modality presents unique characteristics, distributions and scales. This uniqueness can complicate direct comparisons using methods such as MS-SSIM, rendering them less meaningful. Therefore, for measuring the quality of alignment in this context, we need to use an alternative proxy, distinct from MS-SSIM.

In our multimodal consistency loss computation, we use the latent representations derived from the landmark detector. When aligning a sample from modality A with a sample from modality B, we run both samples through the landmark detector and obtain the activations of the first layer, represented as *Z*_A_ and *Z*_B_. We then gauge their similarity using the r.m.s.e., termed the inter-consistency loss:

$${L}_{\mathrm{inter}}={\mathrm{r.m.s.e.}}({Z}_{\mathrm{A}},{Z}_{\mathrm{B}})$$

Moreover, we calculate intra-modality consistency by aligning samples within the same modality and leveraging MS-SSIM for loss computation. This intra-modality consistency mirrors the consistency loss outlined in the previous sections, where one section is registered to another within the same modality:

$${L}_{\mathrm{intra}}={\mathrm{MS-SSIM}}\left({Y}_{\mathrm{registered}},{X}_{\mathrm{target}}\right)$$

Analogous to previous sections, our base loss involves registering a noisy variant of a sample with another perturbed version of the same sample:

$${L}_{\mathrm{base}}={\mathrm{MS-SSIM}}\left({X}_{\mathrm{registered}},{X}_{\mathrm{target}}\right)$$

The final cost function amalgamates the base loss, inter-consistency loss and intra-consistency loss. These are scaled by factors of 10 and 0.1, respectively, as determined empirically:

$${L}_{\mathrm{total}}={L}_{\mathrm{base}}+10 \times {L}_{\mathrm{intra}}+0.1 \times {L}_{\mathrm{inter}}$$

We followed the same protocol for the learning rate schedule, perturbation parameters, batch size and stopping criteria, as detailed in the section regarding runtime experiments. As for Visium data, we maintained the same preprocessing steps described in the earlier section.

### Reporting summary

Further information on research design is available in the Nature Portfolio Reporting Summary linked to this article.

## Online content

Any methods, additional references, Nature Portfolio reporting summaries, source data, extended data, supplementary information, acknowledgements, peer review information; details of author contributions and competing interests; and statements of data and code availability are available at 10.1038/s41592-024-02199-5.

### Supplementary information

Supplementary Information Supplementary Fig. 1.

Reporting Summary

## Data Availability

All experimental data and models are available on figshare at https://figshare.com/projects/ELD/167318. However, the 3D modeling data can be found separately at https://etsin.fairdata.fi.

## Code availability

The code used in the experiments of this paper can be found at https://github.com/ekvall93/ELD.
