# Supplementary material for: Spatial landmark detection and tissue registration with deep learning
Source: Nat Methods. 2024 Mar 4;21(4):673–9. doi: 10.1038/s41592-024-02199-5 (PMC11009106; doi:10.1038/s41592-024-02199-5)
Supplement: Supplementary file 1 — Supplementary Fig. 1. [file 41592_2024_2199_MOESM1_ESM.pdf]

nature methods

Article

https://doi.org/10.1038/s41592-024-02199-5

# Spatial landmark detection and tissue registration with deep learning

In the format provided by the authors and unedited

# Supplementary Information

## Supplementary Figures

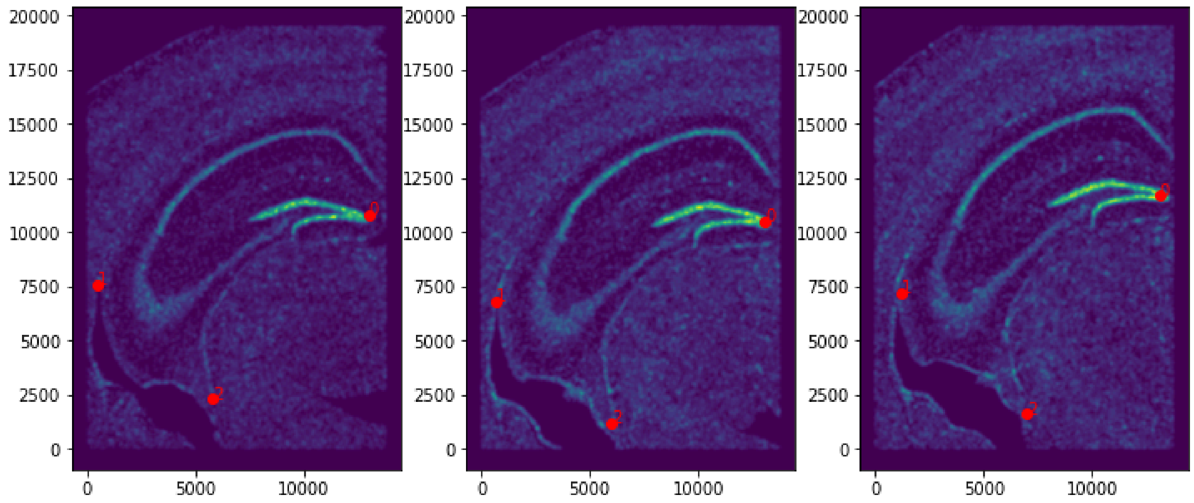


**Supplementary Figure 1:** Manually annotated landmarks used for STAlign benchmark.
