# Supplementary material for: Spatial landmark detection and tissue registration with deep learning
Source: Nat Methods. 2024 Mar 4;21(4):673–9. doi: 10.1038/s41592-024-02199-5 (PMC11009106; doi:10.1038/s41592-024-02199-5)
Supplement: Supplementary file 2 — Reporting Summary [file 41592_2024_2199_MOESM2_ESM.pdf]

nature portfolio

Corresponding author(s): Markus Ekvall

Last updated by author(s): Jan 17, 2024

Reporting Summary

Nature Portfolio wishes to improve the reproducibility of the work that we publish. This form provides structure for consistency and transparency in reporting. For further information on Nature Portfolio policies, see our Editorial Policies and the Editorial Policy Checklist.

Statistics

For all statistical analyses, confirm that the following items are present in the figure legend, table legend, main text, or Methods section.

n/a	Confirmed	
☐	☒	The exact sample size (*n*) for each experimental group/condition, given as a discrete number and unit of measurement
☐	☒	A statement on whether measurements were taken from distinct samples or whether the same sample was measured repeatedly
☒	☐	The statistical test(s) used AND whether they are one- or two-sided<br>*Only common tests should be described solely by name; describe more complex techniques in the Methods section.*
☒	☐	A description of all covariates tested
☒	☐	A description of any assumptions or corrections, such as tests of normality and adjustment for multiple comparisons
☐	☒	A full description of the statistical parameters including central tendency (e.g. means) or other basic estimates (e.g. regression coefficient) AND variation (e.g. standard deviation) or associated estimates of uncertainty (e.g. confidence intervals)
☒	☐	For null hypothesis testing, the test statistic (e.g. *F*, *t*, *r*) with confidence intervals, effect sizes, degrees of freedom and *P* value noted<br>*Give P values as exact values whenever suitable.*
☒	☐	For Bayesian analysis, information on the choice of priors and Markov chain Monte Carlo settings
☒	☐	For hierarchical and complex designs, identification of the appropriate level for tests and full reporting of outcomes
☒	☐	Estimates of effect sizes (e.g. Cohen's *d*, Pearson's *r*), indicating how they were calculated

Our web collection on statistics for biologists contains articles on many of the points above.

Software and code

Policy information about availability of computer code

Data collection	The data utilized in this study underwent preprocessing and was readily accessible from the following articles:<br><br>"A Landmark-based Common Coordinate Framework for Spatial Transcriptomics Data" by Andersson, A. et al.<br>"Comparative analysis of tissue reconstruction algorithms for 3D histology" by Kartasalo, K. et al.<br>"Spatial Multimodal Analysis of Transcriptomes and Metabolomes in Tissues" by Vicari, M. et al.<br>"Optimizing Xenium In Situ data utility by quality assessment and best practice analysis workflows" by Salas, S. M. et al.<br>"Object landmark discovery through unsupervised adaptation" by Sanchez, E. & Tzimiropoulos, G.<br>For additional preprocessing needs, the following tools were used:<br><br>- scanpy==1.9.6 for processing expression data.<br>- opencv-python==4.6.0.66 for image processing<br>- scikit-learn==1.3.2 for PCA dimensionality reduction.<br>- elasticdeform==0.5.0 for elastic noise generation<br>- scikit-image==0.22.0 to make background black when needed
Data analysis	You can find ELD at the following GitHub repository: https://github.com/ekvall93/ELD.<br><br>ELD incorporates these specific packages:<br><br>- torch==1.9.0 as its deep learning library.<br>- piqa==1.2.2 for MS-SSIM loss.

- torchgeometry==0.1.2 for extracting landmarks from heatmaps.
- kornia==0.6.2 for Homography and part of the TPS calcuations.

For comparative analysis, STAlign version 1.0.0 was utilized.

For manuscripts utilizing custom algorithms or software that are central to the research but not yet described in published literature, software must be made available to editors and reviewers. We strongly encourage code deposition in a community repository (e.g. GitHub). See the Nature Portfolio guidelines for submitting code & software for further information.

Data

Policy information about availability of data

All manuscripts must include a data availability statement. This statement should provide the following information, where applicable:

- Accession codes, unique identifiers, or web links for publicly available datasets
- A description of any restrictions on data availability
- For clinical datasets or third party data, please ensure that the statement adheres to our policy

All experimental data and models are available on Figshare at https://figshare.com/projects/ELD/167318. However, the 3D modeling data can be found separately on etsin.fairdata.fi

Human research participants

Policy information about studies involving human research participants and Sex and Gender in Research.

Reporting on sex and gender	Not available
Population characteristics	Not available
Recruitment	Every participant was a minimum of 18 years old, spoke Swedish proficiently, and self-identified as being in good health and mentally stable, as confirmed by the attending midwife. Those who ended their pregnancy due to any medical or psychiatric conditions were excluded. The medical team that provided information to the participants and carried out the abortion procedures did not engage in any other aspect of this study.
Ethics oversight	The Regional Ethical Review Board in Stockholm and the National Board of Health and Welfare granted approval for the use of human developmental heart tissue in the study. The acquisition of the tissue, along with the data processing, complied with the ethical guidelines set forth by the Helsinki Convention (Dnr: 2:9/2015). The use of human foetal material from the elective routine abortions was approved by the Swedish National Board of Health and Welfare, and the analysis using this material was approved by the Swedish Ethical Review Authority (2018/769-31). The human developmental heart tissue, employed in this study, was obtained from medical abortions carried out at the Department of Gynecology, Danderyd Hospital, and Karolinska Huddinge Hospital. All the patients involved gave their informed consent in written form.

Note that full information on the approval of the study protocol must also be provided in the manuscript.

Field-specific reporting

Please select the one below that is the best fit for your research. If you are not sure, read the appropriate sections before making your selection.

☒ Life sciences ☐ Behavioural & social sciences ☐ Ecological, evolutionary & environmental sciences

For a reference copy of the document with all sections, see nature.com/documents/nr-reporting-summary-flat.pdf

Life sciences study design

All studies must disclose on these points even when the disclosure is negative.

Sample size	Mouse olfactory bulb samples: 12 samples; mouse brain coronal: 3 samples; Mouse prostate: 260 samples; Human Developing Heart: 4 samples; mouse striatum: 3 samples.<br><br>The selection of the number of samples was based on the aim to replicate experiments from previous articles, ensuring comparable benchmarks.<br><br>For the new benchmark against STAlign we used the maximum number of replicates available as described in the article "Optimizing Xenium In Situ data utility by quality assessment and best practice analysis workflows" by Salas, S. M. et al. By maintaining identical conditions for both ELD and STAlign we ensured a fair comparison.
Data exclusions	No exclusion

Replication	All benchmarks in the study were replicated by employing the original codebases of the methods being compared.
Randomization	We used that same randomization on the benchmark dataset to have consistent and fair benchmarking result
Blinding	We are not doing any clinical trails in this article

Reporting for specific materials, systems and methods

We require information from authors about some types of materials, experimental systems and methods used in many studies. Here, indicate whether each material, system or method listed is relevant to your study. If you are not sure if a list item applies to your research, read the appropriate section before selecting a response.

Materials & experimental systems

n/a	Involved in the study
☒	☐ Antibodies
☒	☐ Eukaryotic cell lines
☒	☐ Palaeontology and archaeology
☐	☒ Animals and other organisms
☒	☐ Clinical data
☐	☒ Dual use research of concern

Methods

n/a	Involved in the study
☒	☐ ChIP-seq
☒	☐ Flow cytometry
☒	☐ MRI-based neuroimaging

Animals and other research organisms

Policy information about studies involving animals; ARRIVE guidelines recommended for reporting animal research, and Sex and Gender in Research

Laboratory animals	Mouse
Wild animals	Don't apply
Reporting on sex	Don't apply
Field-collected samples	Don't apply
Ethics oversight	No ethical oversight. Use publicly available data used in from other articles

Note that full information on the approval of the study protocol must also be provided in the manuscript.

Dual use research of concern

Policy information about dual use research of concern

Hazards

Could the accidental, deliberate or reckless misuse of agents or technologies generated in the work, or the application of information presented in the manuscript, pose a threat to:

No	Yes
☒	☐ Public health
☒	☐ National security
☒	☐ Crops and/or livestock
☒	☐ Ecosystems
☒	☐ Any other significant area

Experiments of concern

Does the work involve any of these experiments of concern:

No	Yes	
☒	☐	Demonstrate how to render a vaccine ineffective
☒	☐	Confer resistance to therapeutically useful antibiotics or antiviral agents
☒	☐	Enhance the virulence of a pathogen or render a nonpathogen virulent
☒	☐	Increase transmissibility of a pathogen
☒	☐	Alter the host range of a pathogen
☒	☐	Enable evasion of diagnostic/detection modalities
☒	☐	Enable the weaponization of a biological agent or toxin
☒	☐	Any other potentially harmful combination of experiments and agents
